# Human tauopathy strains defined by phosphorylation in R1-R2 repeat domains of tau

**DOI:** 10.1186/s40478-023-01664-0

**Published:** 2023-10-27

**Authors:** Ethan D. Smith, Quan Vo, Benoit I. Giasson, David R. Borchelt, Stefan Prokop, Paramita Chakrabarty

**Affiliations:** 1 Center for Translational Research in Neurodegenerative Disease, University of Florida, 1275 Center Drive, BMS J484, Gainesville, FL 32610 USA; 2 Department of Neuroscience, University of Florida, Gainesville, FL 32610 USA; 3 McKnight Brain Institute, University of Florida, Gainesville, FL 32610 USA; 4 Department of Pathology, Immunology & Laboratory Medicine, University of Florida, Gainesville, FL 32610 USA; 5 Fixel Institute for Neurological Diseases, University of Florida, Gainesville, FL 32610 USA

**Keywords:** Post-translational modification, Seeding, Alzheimer’s disease, Progressive supranuclear palsy

## Abstract

Distinctive post-translational modifications (PTM) characterize tau inclusions found in tauopathy patients. Using detergent-insoluble tau isolated from Alzheimer’s disease (AD-tau) or Progressive Supranuclear Palsy (PSP-tau) patients, we provide insights into whether phosphorylation of critical residues determine templated tau seeding. Our initial data with phosphorylation-ablating mutations (Ser/Thr → Ala) on select sites of P301L tau showed no changes in seeding efficacy by AD-tau or PSP-tau. Interestingly, when specific sites in the R1-R2 repeat domains (Ser262/Thr263/Ser289/Ser305) were mutated to phosphorylation-mimicking amino acid Glu, it substantially reduced the seeding efficiency of AD-tau, but not PSP-tau seeds. The resultant detergent-insoluble tau shows deficient phosphorylation on AT8, AT100, AT180 and PHF1 epitopes, indicating inter-domain cooperativity. We further identify Ser305 as a critical determinant of AD-tau-specific seeding, whereby the phospho-mimicking Ser305Glu tau abrogates seeding by AD-tau but not PSP-tau. This suggests that phosphorylation on Ser305 could be related to the formation of disease-specific tau strains. Our results highlight the existence of a phospho-PTM code in tau seeding and further demonstrate the distinctive nature of this code in 4R tauopathies.

**Supplementary Information:**

The online version contains supplementary material available at 10.1186/s40478-023-01664-0.

## Introduction

Intraneuronal inclusions of hyper-phosphorylated protein tau are a major pathological feature of numerous neurodegenerative diseases, such as Alzheimer’s disease (AD), Progressive Supranuclear Palsy (PSP), Cortico-basal degeneration (CBD) and others [1, 2]. Current modeling studies show that misfolded tau can self-template native cellular tau inducing a pathologic cascade whence these newly-generated tau seeds transfer along neuroanatomic connections, leading to dissemination of tau pathology [3–5]. It has been suggested that post-translational modifications (PTM) on tau, phosphorylation in particular, play a key role in promoting misfolding and aggregation of tau [6], though this remains somewhat disputed [7]. In our study, we asked the specific question: does presence of disease-associated PTMs influence tau’s propensity to be self-templated?

Hyperphosphorylation of tau leads to both loss of function and gain of function properties for tau protein, including loss of microtubule binding, altered axonal transport and increased propensity to aggregate [8–10]. A recent study has shown that tau seeds derived from human brains display differential phosphorylation patterns that imparts high or low seeding efficiencies [11], which is indicative of molecular diversity inherent in these individuals. These and similar observations have led to the proposition of the existence of tau strains [12]. Recent studies have also shown that presence of specific tau phosphorylation epitopes on soluble tau can distinguish AD from other related dementias [13]. However, no studies have addressed whether pre-existing phosphorylation patterns on soluble naïve tau regulate its efficiency to be templated by tau seeds derived from patient brains, i.e., does the strain concept extend to the phosphorylation patterns on host soluble tau and are there disease-specific phosphorylation patterns on host tau that determine what disease-specific strain will predominate?

A recent study conducted in a cross section of elderly healthy and AD patients has identified multiple Ser/Thr residues in tau that are most frequently phosphorylated at early stages in individuals with AD dementia [14]. Here, we have investigated whether these phosphorylation sites are crucial to determining seeding efficiency of AD (AD-tau) or PSP (PSP-tau) brain derived tau seeds, using HEK 293 T cells expressing 0N/4R P301L tau as seeding recipients. We used a mutagenesis approach where we clustered phosphorylation-substitution mutations on 19 identified sites to create 5 variants on the 0N/4R P301L tau sequence on the basis of spatial proximity (3 in the proline rich region (PRR); 1 in repeat domains R1-R2; 1 in C-terminal region). Using a HEK293T cell assay, we measured the extent of donor tau-seeded aggregation by normalizing the tau levels in the insoluble cellular fraction to the tau levels present in both soluble and insoluble cell fractions. Here, we show that phospho-mimicking substitutions (Ser/Thr → Glu; referred to as ‘Phospho-Plus’ henceforth) on specific epitopes in the R1-R2 repeat domains (S262/T263/S289/S305) restrict the seeding ability of AD-tau, but not PSP-tau. The resultant seeded tau displayed differential phosphorylation patterns in the PRR domain and C terminal region, indicating that altered phosphorylation in the repeat domain also affects other major AD-typical phosphorylation sites. Phospho-Plus substitutions in the PRR domain of tau did not influence seeding activity of either AD-tau or PSP-tau. Phosphorylation ablating mutations (Ser/Thr → Ala; referred to as ‘Phospho-Null’ henceforth) on these same sites did not show any altered efficacy on seeding. We further identify phosphorylation of S305 as an AD-discriminatory tau seeding epitope. Our observations, replicated in 6 unique AD-tau and 6 PSP-tau seed sources, highlight the existence of a phospho-PTM code on host cellular tau and further demonstrates the distinctive nature of this code in AD and PSP.

## Materials and methods

### Generation of mutant tau phospho-substituted constructs

Phospho-site substitutions were generated in the 0N4R P301L tau backbone under contract with Genscript. The backbone vector is CTR0 containing hybrid chicken β-actin promoter and bovine poly A and carrying Ampicillin resistance. All constructs were Sanger sequenced and restriction enzyme digested for sequence accuracy and validation. pcDNA3.1( +) encoding for 0N4R P301L tau and 0N4R P301L/S305E tau was reported earlier [15].

### Cell culture and transfection

HEK293T cells were maintained in Dulbecco’s modified Eagle’s medium (Invitrogen, Carlsbad, CA) supplemented with 10% fetal bovine serum (FBS) and 100 U/ml penicillin/100 μg/ml streptomycin at 37 °C and 5% CO2. For transfections, cells were plated on 24-well polystyrene plate with 1 mL of media. For cellular transfection, once cells reached ~ 60% confluency, 0.4 μg of plasmid DNA expressing 0N4R tau was combined with Lipofectamine 3000 reagents (Thermo Fisher Scientific) according to the manufacturer’s instructions. This mixture was incubated at room temperature for 10–15 min before adding dropwise to the media in each well and placed in the incubator for 30 min. For seeding, 0.5 µg of patient derived insoluble tau was incubated with Lipofectamine reagent for 20 min and added to the cells. Cells were harvested 48 h after transfection and fractionated for detergent soluble and insoluble protein.

### Biochemical cellular fractionation of HEK cells

Cells were harvested in 50 μL of High Salt Buffer (50 mM Tris–HCl, pH 7.4, 250 mM NaCl, 2 mM EDTA, 1% Triton X-100, 20 mM NaF) and a cocktail of protease and phosphatase inhibitors (Pierce #A32959) (aprotinin, bestatin, E-64, leupeptin, sodium fluoride, sodium orthovanadate, sodium pyrophosphate, β-glycerophosphate, and EDTA). Samples were sedimented at 150,000 × g for 30 min at 4 °C and the supernatants collected. To ensure supernatant removal, pellets were washed and sedimented again. Supernatants were removed and the pellets were resuspended in 50 μL of High salt buffer. 5X SDS sample buffer (final concentration of 250 mM Tris–Cl, pH 6.8, 5% β-Mercaptoethanol, 0.02% Orange G, 10% SDS, 30% glycerol) was added to the collected supernatants and resuspended pellets, referred to as the Triton-soluble and Triton-insoluble fractions. To resuspend the pellet, Triton-insoluble samples were probe-sonicated. These samples were then heated at 90 °C for 10 min and stored in the -80 °C until SDS-PAGE.

The primary outcome of the study is measured as % tau aggregation. The % tau aggregation is calculated as [(total tau in detergent insoluble fraction)/(total tau in detergent soluble fraction + total tau in detergent insoluble fraction)]*100. Normalized AT8 was calculated as [AT8 signal in detergent-insoluble seeded fraction/AT8 signal in detergent-soluble seeded fraction]. Tau expression was calculated as [Total (unseeded) tau in detergent soluble fraction/GAPDH].

### Purification of insoluble Tau from AD and PSP brains

Human brain tissues from six AD cases, seven PSP cases, and two non-demented control cases (all cases from the UF Brain Bank brain bank) were selected for this study (Additional file 2: Table S1). All cases were diagnosed based on accepted neuropathology criteria. Purification of pathological, insoluble tau from the temporal cortex of AD and non-demented control cases, or lentiform nucleus of PSP cases were performed as previously described [16]. Briefly, for the purification of AD-tau and PSP-tau, 50–100 mg of temporal cortical gray matter or lentiform nucleus (respectively) was homogenized in nine volumes (v/w) of high-salt buffer (10 mM Tris with 0.8 M NaCl, pH7.4) with 0.1% Sarkosyl and 10% sucrose added, and centrifuged at 10,000 × g for 10 min at 4 °C. Pellets were re-extracted twice using the same high-salt buffer and the supernatants from all three extractions were filtered and pooled. Additional Sarkosyl was added to the pooled supernatants to reach 1% and the samples were nutated for 1 h at 37 °C. The samples were centrifuged at 150,000 × g for 60 min at 15 °C and the resulted 1% Sarkosyl-insoluble pellets containing pathological tau were resuspended in PBS. The resuspended Sarkosyl-insoluble pellets were further purified by a brief sonication using a handheld probe (Qsonica), followed by centrifugation at 100,000 × g for 30 min at 4 °C. The pellets were resuspended in PBS at 1/2 to 1/5 of the precentrifugation volume, sonicated, and spun at 10,000 × g for 30 min at 4 °C to remove large debris.

The final purified supernatants containing insoluble, pathological tau, and are identified as AD-tau and PSP-tau in subsequent experiments. The final fraction was analyzed by Western blotting and sandwich ELISA for tau (Invitrogen #KHB0041). The sandwich ELISA and Western blotting for tau in the final supernatant were used for estimates of tau concentration. The final supernatants were also analyzed by bicinchoninic acid (BCA) assay (Fisher) for total protein concentration.

### Microtubule binding assay

Cells were lysed in 100 μl of PEM buffer (80 mM PIPES, pH 6.8, 1 mM EGTA, 1 mM MgCl2) supplemented with 0.1% Triton X-100, 2 mM GTP, 20 μM paclitaxel, and a mix of protease inhibitors as described previously [17]. For the “without paclitaxel” condition, cells we lysed in PEM buffer supplemented with 0.1% Triton X-100, 2 mM GTP, and a mix of protease and phosphatase inhibitors. Cell lysates were incubated in a 37 °C water bath for 30 min and then centrifuged at 100,000 × g for 30 min to pellet mictrotubules (MT). Supernatant was transferred to a new tube, and the pellet (MT fraction with bound proteins) was resuspended in PEM buffer. The pellet fraction was briefly sonicated, and SDS gel loading buffer was added to both fractions. Equivalent amounts of supernatant and pellet were loaded on SDS–polyacrylamide gels for Western blot analysis. Percent MT bound tau was calculated as pellet/(supernatant + pellet) × 100.

### Western blotting

8 μl of triton-soluble and 15 μl of triton-insoluble samples were loaded on 4–20% Tris–Glycine gels (Invitrogen #XP04205) and run for 1.5 h at 100 V. Blots were then transferred to 0.45 μm PVDF membranes (Millipore #IPFL85R) for 2 h at 300mAmps before being blocked in 0.5% Casein for 1 h. Blots were incubated in appropriate primary antibodies overnight as indicated in each figure (Additional file 2: Table S2), and then incubated with IRDye-labeled secondary antibodies and scanned using an ODY-2536 Imager.

### Immunocytochemistry

HEK293T cells were washed 3 times with PBS and fixed with 10% formalin for 15 min at RT. Staining was performed with 0.0125% (wt/vol) ThioS in 50% ethanol for 3 min. ThioS was differentiated in 50% ethanol in PBS for 30 s. Coverslips were mounted using ProLong Glass Antifade Mountant with NucBlue Stain (Invitrogen #P36981) to label cell nuclei. A Keyence microscope was used to acquire immunofluorescence images. For the quantification of ThioS positivity, 20X images were taken at random along the coverslip and manually counted using ImageJ.

### Cell viability

Confluent HEK cells in a 24-well plate were used to test viability of cells via alamarBlue (Invitrogen #DAL1025). Briefly, each well received alamarBlue reagent and a well with only media was used as a blank control (the reagent is at 10x, therefore 100 μl was used to add to 1 mL of media/well). Cells were incubated for different time points to track viability (2 h, 4 h, and 24 h) in which 100 µl of media was drawn from each condition and stored in a clear, round-bottom 96-well plate and sealed until all timepoints were collected. After, the plate was run on a plate reader and fluorescence excitation/emission was detected around 540–570/580–610 nm and absorbance at 570 nm.

### Statistical analysis

Western blot signals were quantified based on densitometric analysis using ImageJ. Manual cell counting was performed using ImageJ. For statistical comparisons, we performed both one-way and two-way analysis of variance (ANOVA) with either a Dunnett’s or a Bonferroni’s multiple comparisons test, respectively, to compare each group to the control.

## Results

### Detergent insoluble AD-tau and PSP-tau seeds induce P301L tau to form aggregates in HEK293T cells

We isolated detergent-insoluble tau seeds using a serial fractionation protocol from brains donated by AD, PSP and age-matched cognitively unimpaired individuals (Additional file 1: Fig. S1a-b, Table S1). Because previous data has shown heterogeneity in among patients [11], we used cortical tau from 6 AD patients and lentiform nuclear tau from 6 PSP patients in our study (Table S1). We confirmed the presence of tau in AD-tau and PSP-tau seed fractions using immunoblotting (Additional file 1: Fig. S1c, g) and ELISA (Additional file 1: Fig. S1d, h). We used a HEK293T cell-based model, which has been used reproducibly in our labs in previous studies [18–21] to examine the capability of these tau seeds to template 0N/4R WT human tau or 0N/4R P301L tau. We found that detergent-insoluble seeds derived from AD (AD-tau) and PSP (PSP-tau) patients could induce formation of AT8-positive detergent-insoluble tau in cells expressing 0N/4R P301L tau (Additional file 1: Fig. S1e, i). Neither AD-tau nor PSP-tau seeds induced detergent-insoluble tau in cells expressing 0N/4R WT tau or Green Fluorescent Protein (GFP) (Additional file 1: Fig. S1f, j). We next performed an optimization step for AD-tau and PSP-tau seeds in this assay and found that as little as 0.0625 µg of S3 fraction (AD-tau) and 0.312 µg of S3 fraction (PSP-tau) was sufficient to induce AT8-positive detergent-insoluble tau (Additional file 1: Fig. S2a-b) and that transfection with 0.4 µg of P301L tau DNA was optimal to observe seeding (Additional file 1: Fig. S2c-d) with no cell death (Additional file 1: Fig. S2e). The result of these initial studies produced a reliable model system to assess tau seeding using donor-derived tau seeds.

In ADRDs, tau undergoes phosphorylation simultaneously at multiple sites [8] and recent phospho-proteomic analysis suggests that progressive phosphorylation on specific sites precede misfolding and aggregation into NFT [14]. From this study, we identified 6 phosphorylation sites that are modified in pre-symptomatic normal individuals, 7 additional sites that are phosphorylated in a subset of individuals that are pre-symptomatic or definite AD, and 6 more sites that are phosphorylated in definite AD cases (total 19 sites, numbered according to 2N/4R tau: Additional file 1: Fig.S3a). This led us to hypothesize that ablating phosphorylation on these sites could prevent tau from being misfolded in the initial seeding phase. To examine whether phosphorylation on these sites influence self-templating properties of misfolded tau, we introduced Ala substitutions on all 19 sites of phosphorylation spanning across the Proline rich region (PRR), microtubule binding region (MTBR) and C terminal domain, referred to as Complete Phospho-Null (CPN) construct (Additional file 1: Figs. S3a). We also divided these 19 sites into 5 spatially contiguous phospho-substitution sites spanning across human P301L 0N/4R tau sequence (Additional file 1: Fig. S3a). Numbering these 1 through 5, we mutated the Ser/Thr sites on these clusters to phospho-nullifying amino acid Alanine (Ala). The rationale was based on earlier precedence of using such combined mutations to examine microtubule binding, aggregation and seeding properties of tau [6, 20, 22]. We established that the relative levels of tau expression are similar between the different Phospho-Null Variants 1–5 (Additional file 1: Fig. S3a).

To test our hypothesis that ablating phosphorylation would reduce seeding, we first tested whether AD-tau seeds or PSP-tau seeds would template the CPN construct transfected into HEK293T cells, with P301L tau as the positive control and seeds from healthy patients as negative controls (Additional file 1: Fig. S3b-o). We observed that the CPN construct produced insoluble tau when seeded by six different AD-tau (Additional file 1: Fig. S3b-e) and six PSP-tau seed preparations (Additional file 1: Fig. S3i-l). Homogenates from healthy controls did not induce aggregation of P301L tau (Additional file 1: Fig S3c; Patient ID # 1 and 2). As a positive control, unmodified P301L tau was readily seeded by AD-tau seeds and PSP-tau seeds (Additional file 1: Fig S3c, j; #B). Only seeded P301L tau was reactive to AT8 because one of the Ala mutations destroyed the AT8 epitope on the CPN construct (Additional file 1: Fig. S3e, l). We also confirmed that when we tested on individual Phospho-Null Variants 1 through 5, both AD patient-derived tau seeds (Additional file 1: Fig. S3f-h) and PSP patient-derived tau seeds (Additional file 1: Fig. S3m-o) showed equivalent tau seeding efficiency compared to the other variants. These somewhat surprising findings indicate that biochemically mimicking phosphorylation ablation of tau at these 19 early sites does not modulate seeded tau aggregation in cell models.

### Phospho-Plus substitutions in the R1-R2 repeat domains alter seeding efficiency of AD-tau

**Fig. 1:**
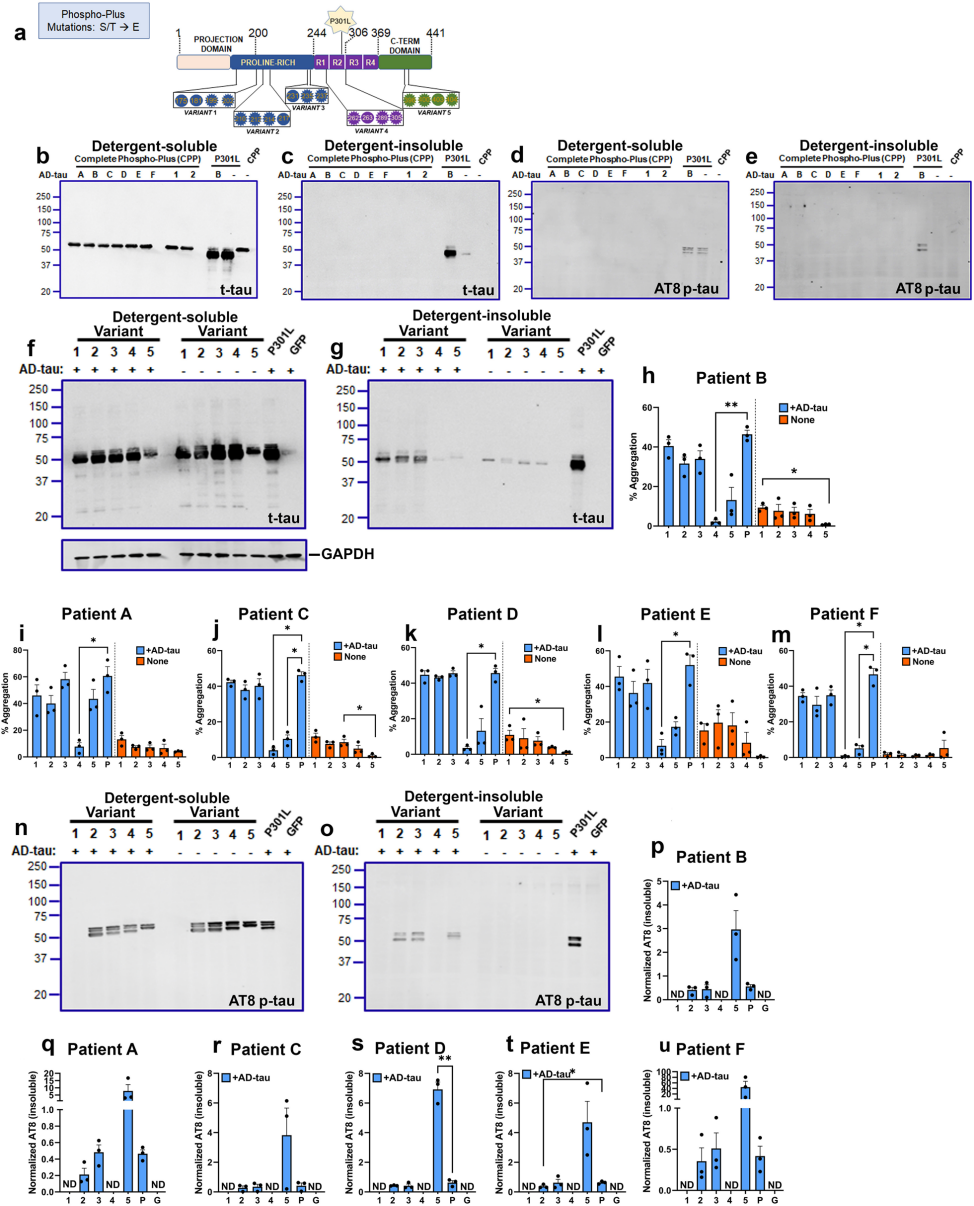
AD-tau seeds show differential effects on Phospho-Plus tau variants.** a.** Schematic depiction of Phospho-Plus Ser/Thr → Glu tau variants generated on human 0N/4R P301L mutant tau. All numbers correspond to 2N/4R tau. R1-R4 depict microtubule-binding repeat regions. **b–e.** Representative immunoblot of the Complete Phospho-Plus (CPP) construct with all 19 sites mutated to Glu and seeded with Patients A-F or two healthy controls (1, 2) shown. P301L tau seeded with Patient B or left unseeded are also shown. Total tau (t-tau) and AT8-tau (p-tau) shown from detergent-soluble (**b**, **d**) or insoluble (**c**, **e**) cell lysates. **f–u**. AD-tau ( +) or unseeded (-) HEK293T cells transfected with different tau variants were fractionated into detergent-soluble and detergent-insoluble lysates and probed for total tau (**f**–**g**, t-tau) or AT8 (n–o, p-tau). Quantitation of % aggregation (insoluble/[soluble + insoluble]*100) for each AD-tau variant are shown (h-m). AT8 levels in detergent insoluble lysates has been normalized and shown (p-u). GAPDH is the loading control for detergent-soluble fraction. Broken lines (**h**–**m**) depict statistical tests done separately within groups of seeded or non-seeded tau variants. Numbers 1–5 denote the tau Variants; ‘P’, P301L tau; ‘G’, GFP; ‘ND’, not detected. Representative blots for all other patients not depicted here (A, C, D, E and F) are shown in Figure S4.

To complement the previous part of this study, we further designed Phospho-Plus tau constructs by mutating Ser/Thr sites to the phospho-mimicking Glutamate (Glu) amino acid. We created the Complete Phospho-Plus (CPP) construct with 19 substitutions as well as individual Variants 1–5, with Glu substitutions as indicated (Fig. 1a). Surprisingly, when we used the CPP construct, we found that none of the AD-tau seeds from 6 different patients induced tau seeding (Fig. 1b–c), which was confirmed by an absence of AT8 signal (Fig. 1d–e).

To refine which phosphorylation sites may be responsible for our observed outcome, we used the five Phospho-Plus Variants containing phospho site Glu substitutions in spatially contiguous clusters (Fig. 1; Additional file 1: Additional file 1: Fig. S4). When we performed the AD-tau seeding assay with these variants, we found that Variants 4 and 5 were less susceptible to seeding than variants 1–3 (Fig. 1f–h). Variant 4 (containing Phospho-Plus mutations in the R1-R2 region) showed lower seeded tau levels in the detergent-insoluble fraction compared to seeded P301L tau when seeded with AD Patient B (Fig. 1h; *p* < 0.01). Indeed, using multiple donor AD-tau and executing three replicates per patient, Variant 4 appears to be consistently less prone to seeded aggregation (Fig. 1i–m; additional experimental and biological replication blots in Additional file 1: Fig. S4), which indicates a robust loss of self-templating ability of Variant 4 across patients. Using Thioflavin S staining, we confirmed that the levels of tau ‘amyloid’ was indeed reduced in Variant 4 seeded with AD-tau relative to Variant 5 and parent P301L tau seeded with AD-tau from Patient B (Additional file 1: Fig. S5). A similar pattern emerged when soluble and insoluble fractions of AD-tau seeded HEK293T cells were probed with AT8 (Fig. 1n–p; additional experimental and biological replicates: Fig. 1q–u; Additional file 1: Fig. S4). When we normalized AT8 levels in insoluble fraction to total insoluble tau, we confirmed that the seeded Variant 4 selectively showed no detectable phosphorylation on Ser202/Thr205 epitope (Fig. 1p–u, Additional file 1: Fig. S4). Notably, Variant 1 did not show any AT8 reactivity as this isoform contains substitution on the AT8 epitope (Ser202/Thr205).

The levels of expression for the Phospho-Plus tau variants was comparable with each other (Additional file 1: Fig. S4h). Notably, seeding of cluster Variant 5 with AD-tau was variable. Compared to parent P301L tau, seeded Variant 5 resulted in reduced levels of total tau in the detergent-insoluble cellular fractions (Fig. 1f–m; Patient C and Patient F**:** *p < 0.05). On the other hand, seeds from Patient A showed robust aggregation and seeds from Patients B, D and E showed reduced aggregation (Fig. 1i–m). This would be consistent with previous observations that tau seeding activity displays heterogeneity between different AD donors and this could be related to the phosphorylation profile of seed-competent tau [11, 23]. Interestingly, we noted that Variant 5 consistently showed higher AT8 levels comparable to Variants 2 and 3, as well as parent P301L tau (Fig. 1p–u; *p* < 0.01 for Patient D), in spite of its overall lower aggregation propensity detected by total tau antibody. This would suggest some dissociation between aggregation propensity and AT8 site phosphorylation when tau is phosphorylated in the C-terminus.

### Phospho-Plus PTM isoforms do not significantly alter seeding efficiency of PSP-tau seeds

**Fig. 2:**
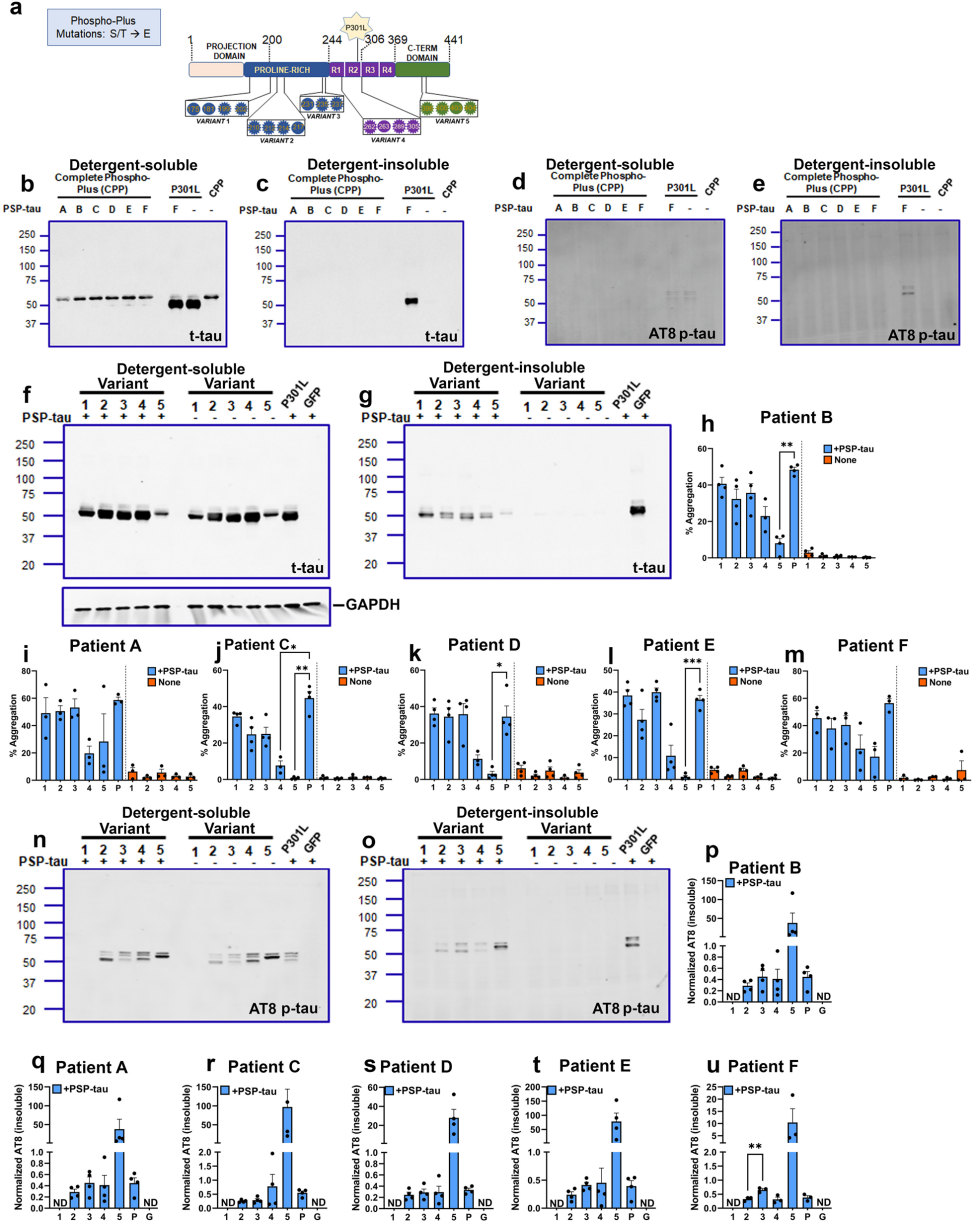
Analysis of seeding efficiency of PSP-tau on Phospho-Plus tau variants.** a.** Schematic depiction of Phospho-Plus Ser/Thr → Glu tau variants generated on human 0N/4R P301L mutant tau. All numbers correspond to 2N/4R tau. R1-R4 depict microtubule-binding repeat regions. **b–e.** Representative immunoblot of the Complete Phospho-Plus (CPP) construct with all 19 sites mutated to Glu and seeded with Patients A-F shown. P301L tau seeded with Patient F or left unseeded are also shown. Total tau (t-tau) and AT8-tau (p-tau) shown from detergent-soluble (**b**, **d**) or insoluble (**c**, **e**) cell lysates. **f–u.** PSP-tau ( +) or unseeded (−) HEK293T cells transfected with different tau variants were fractionated into detergent-soluble and detergent-insoluble lysates and probed for total tau (f-g, t-tau) or AT8 (n–o, p-tau). Additional replicate blots are shown in Fig. S6. Quantitation of % aggregation (insoluble/[soluble + insoluble]*100) for each variant are shown (h-m). AT8 levels in detergent insoluble lysates has been normalized and shown (p-u). GAPDH is the loading control for detergent-soluble fraction. Broken lines (**h**–**m**) depict statistical tests done separately within groups of seeded or non-seeded tau variants. Numbers 1–5 denote the tau Variants; ‘P’, P301L tau; ‘G’, GFP; ‘ND’, not detected. Representative blots for all other patients not depicted here (A, C, D, E and F) are shown in Additional file 1: Fig. S6. Relative molecular masses (kDa) are indicated on the left of each blot. N = 3 experimental replicates. 1-way ANOVA with Dunnett’s test; ****P < 0.0001; **p < 0.01, *p < 0.05

To examine if the strain differences between AD-tau and PSP-tau [24, 25] are reflected in the self-templating properties of tau, we next examined if these same Phospho-Plus (Ser/Thr → Glu) variants alter the propensity of PSP-tau in forming tau aggregates (Fig. 2). First, we examined the CPP construct with all 19 sites substituted for Glu. Consistent with our data on AD-tau seeding, the CPP construct was not susceptible to PSP-tau seeds derived from 6 different patients as detected by total tau antibody and AT8 antibody (Fig. 2b–e). We then examined how the 5 individual Phospho-Plus Variants respond to PSP-tau seeds from different patients. First, using soluble fractions of seeded and unseeded HEK293T cells, we found little difference in soluble levels of total tau between parent P301L tau and the Phospho-Plus variants, except for Variant 5 (Fig. 2f–m; Additional file 1: Fig. S6: additional blots from other patients). Additionally, analysis of the insoluble cell fraction showed that Variants 1, 2, 3 and 4 showed equivalent levels of total seeded tau using PSP-tau from different patients (Fig. 2f–m: Additional file 1: Fig. S6 for additional patients and replicate blots). Some heterogeneity was noted in this aspect, especially for PSP Patient C seeds which showed lower insoluble tau when seeding Variant 4 (Fig. 2j; *p* < 0.05). Variant 5 consistently showed reduced seeding efficiency with PSP-tau derived from Patient B (*p*  < 0.05), Patient C (*p*  < 0.01), Patient D (*p*  < 0.05) and Patient E (*p*  < 0.05), with trend for Patients A and F (Fig. 2h-m). AT8 analysis showed that Variants 2–4 showed comparable soluble and insoluble tau levels, relative to P301L tau (Fig. 2n–u; Additional file 1: Fig. S6). Compared with the other variants, Variant 5 showed higher AT8 burden in spite of lower amounts of total tau in the insoluble fraction, though the data did not reach statistical significance (Fig. 2p–u). This observation is consistent with the data from AD-tau seeding (Fig. 1). Overall, notwithstanding some heterogeneity between patients, our data shows that Phospho-Plus Variant 4 can be seeded by PSP-tau, suggesting that the phosphorylation sites contained in this variant potentially differentiate PSP and AD seeding susceptibility, with phosphorylation mimics in this construct inhibiting AD-tau seeding consistently.

### Cooperative phosphorylation profiles of R1-R2 Phospho-Plus variants following tau seeding

**Fig. 3:**
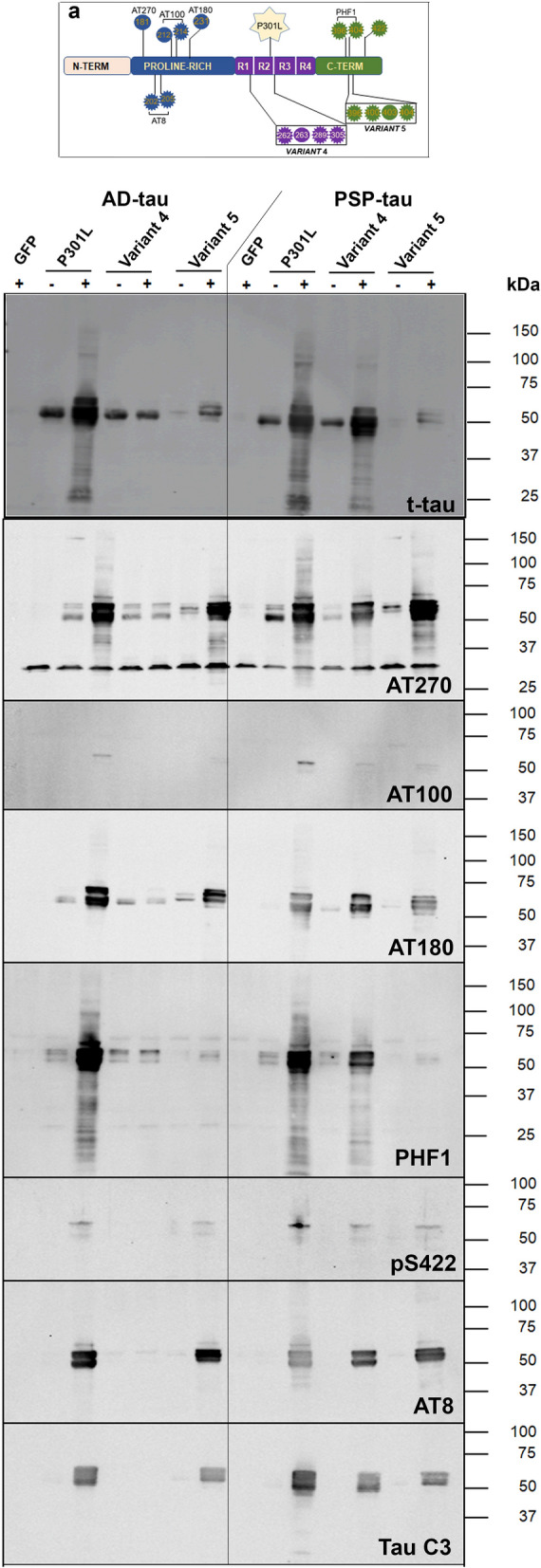
Phosphorylation profile of MTBR-associated tau variants following seeding. **a**. Schematic depiction of Phospho-Plus Ser/Thr → Glu tau variants generated on human 0N/4R P301L backbone and antibody epitopes used to detect tau phosphorylation. **b.** Detergent-insoluble fraction of AD-tau ( +), PSP-tau ( +) or unseeded (–) HEK293T cells transfected with different tau variants and GFP were probed for different epitopes. Relative molecular masses are indicated on the left of each blot. Blots showing technical replicate and detergent-soluble fractions are presented in Additional file 1: Fig. S7 a-d

We wanted to investigate how the phospho-PTM profile on host tau influences the phosphorylation patterns of tau aggregates resulting from seeding [26]. Looking at seeded Phospho-Plus Variant 4 with total tau antibody, we could reproduce our observations that AD-tau does seed Variant 4 less robustly than PSP-tau seeds (Additional file 1: Fig. S7a-d). Using antibodies against phospho-tau epitopes that are distributed in the PRR and C terminal region which were previously identified to be over-represented in AD (Fig. 3a; AT270, AT100, AT180, PHF1, pSer422 and AT8), we found that PSP-tau seeded Variant 4 showed robust phosphorylation on these sites and was identical to P301L tau (Fig. 3b). However, AD-tau seeded Variant 4 showed minimal phosphorylation on these epitopes, especially with no detectable signals for AT8, AT100 and pSer422. Of note, TauC3, a truncated form of tau generated by caspase-3 cleavage at D421 and a precursor of bioactive seeds [27], was present only in PSP-tau seeded Variant 4 but not in AD-tau seeded Variant 4 (Fig. 3b). Because the levels of phosphorylation on AT270, AT8, AT100, AT180, and pSer422 epitopes was equivalent between Variant 5 and P301L tau, this indicates that most of the seeded Variant 5 is highly phosphorylated in spite of total tau levels being lower. This finding suggests that pre-existing phosphorylation on these C terminal epitopes could preferentially drive the emergence of AD-typical phosphorylation pattern following seeding. Notably, we found little to no PHF1 in seeded Variant 5, which is somewhat expected as PHF1 epitope (Ser396/Ser404) overlaps the Phospho-Plus substitutions in Variant 5. We also noted that both AD-tau and PSP-tau resulted in equivalent levels of cleaved TauC3 in seeded Variant 5, but not in seeded Variant 4 seeded with AD-tau (Fig. 3b).

Phospho-PTMs in tau result in altered structure that modify normal function of tau (such as microtubule binding) or add pathological attributes (increased propensity to aggregate) [1, 28]. Because we observed an interesting dichotomy between levels of total tau and phosphorylated tau in Variant 5, we wondered whether Variant 5 was inherently more prone to show reduced microtubule affinity. To assess tau how these Phospho-Plus mutations lead loss of microtubule binding, we performed microtubule binding assay in the presence and absence of paclitaxel, that stabilizes microtubule-tau interaction (Fig. S7i). Both Variant 4 and Variant 5 tau showed lower propensity to bind microtubules, compared to parent P301L tau (Additional file 1: Fig. S7e-f). Additionally, we also confirmed that, in the presence of paclitaxel, both Variant 4 (p < 0.001) and Variant 5 (p < 0.0001) showed lower microtubule binding affinity respectively compared to P301L tau (Additional File 1: Fig. S7g-h).

### Ser305 within MTBR-R2 domain is a specific regulator of tau seeding

**Fig. 4:**
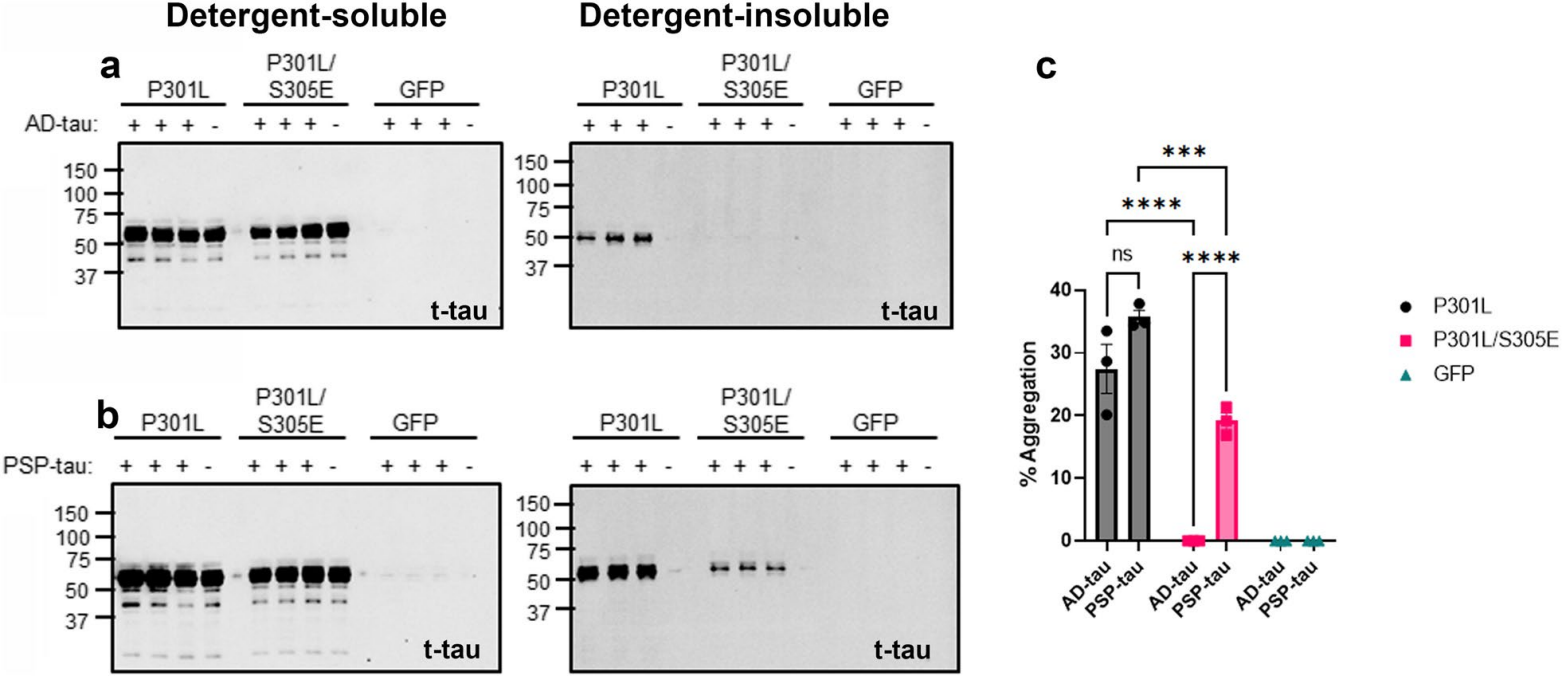
Phospho-Plus mutation on Ser305 abrogate seeding by AD-tau. **a**–**c**. AD-tau ( +), PSP-tau ( +) or unseeded (–) HEK293T cells transfected with P301L, P301L/S305E tau or GFP were fractionated into detergent-soluble and detergent-insoluble lysates and probed for total tau (t-tau). Quantitation of % aggregation (insoluble/[soluble + insoluble]*100) for each variant are shown (**c**). 2-way ANOVA with Bonferroni’s test. ***p < 0.001; **** p  < 0.0001

Having identified that Phospho-Plus substitutions on S262/T263/S289/S305 prevented the ability of AD-tau from seeding P301L tau, we wanted to determine if individual phospho-mimetic epitopes within this variant region could specifically drive this differential seeding outcome. We introduced the phospho-mimetic S305E substitution on P301L tau and we tested the efficiency of AD-tau and PSP-tau in seeding P301L and P301L/S305E tau (Fig. 4). We observed that both AD-tau and PSP-tau could successfully seed P301L tau (Fig. 4a, b; % aggregation quantified in 4c). However, only PSP-tau, but not AD-tau, was able to seed P301L/S305E tau (Fig. 4a, b). This suggests that S305 phosphorylation could be a discriminatory epitope in soluble tau that promotes templating by PSP-tau and is inhibitory for AD-tau templating.

## Discussion

In this study, we set out to investigate whether phospho-substitutions on AD-associated phospho-epitopes would alter seeding efficiency of tau seeds derived from AD and PSP patients. We found that a cluster of four Phospho-Plus mutations in the R1-R2 domains, and specifically the glutamate-substituted S305 epitope within this region, led to ablation of seeding by AD-tau, with PSP-tau retaining seeding activity. We also found that clustered Phospho-Plus substitutions in the C-terminal region led to reduced seeding efficiency by both AD and PSP patients, but AT8 phosphorylation was over-represented in the tau that was seeded. Phospho-Plus substitutions in the PRR region did not lead to altered seeding efficiency by either AD-tau or PSP-tau. This suggests the existence of a phospho-PTM code that influences tau seeding and that tau seeds from different sources show unique templating characteristics. Notwithstanding some level of patient heterogeneity encoded in the seeds, we speculate that differential site-specific phosphorylation events on S305 could initiate nucleation in the tau seeding process uniquely in PSP but not in AD. Our study thus provides a molecular framework to define strain-like characteristics in the context of phosphorylated tau.

Previous studies on seeding have not fully explored how altering the phospho-PTM of the host soluble tau would affect the seeding ability of human brain-derived tau seeds. The broad significance of our study is that it addresses whether select phospho-PTMs on host tau can regulate the initial nucleation of tau fibers when exposed to tau seeds from diseased brains. Several seminal studies have shown that mutant recombinant seeds or human brain-derived tau seeds obtained from neurodegenerative diseases with unique clinical presentations have different seeding and aggregation properties in vitro and in vivo [24, 29–33]. Additionally, a recent study showed that seeding ability of AD-tau (oligomeric seeds) was partially related to the phosphorylation profile of the patient [11]. Using presence of Fluorescence Resonance Energy Transfer (FRET) biosensor activity, this study also identified T231/S235 and pS262 phosphorylation on AD-tau seeds positively correlating with tau seeding, while S198/S199/S202 and S400/T403/S404 correlating negatively [11]. Another study considered whether acetylation, another major tau PTM, could alter tau priming, finding that acetylated K18-tau seeds accelerate tau seeding in that FRET biosensor HEK293 cells [34]. While a direct comparison of our study cannot be done with these reports, collectively these studies certainly identify PTMs on the tau seeds as crucial determinants of tau seeding and aggregation.

Our study adds to growing evidence regarding the complexity of phosphorylation as an intermediate event leading to tau misfolding and aggregation. Many studies have posited that tau needs to be hyperphosphorylated to be assembled into inclusions and that hyperphosphorylation is functionally related to tau dysfunction [6, 35]. Indeed, our initial hypothesis was that phospho-nullifying substitutions on AD-associated sites would lead to abrogation of seeding and tau aggregation. It was surprising that these Phospho-Null substitutions did not influence seeding characteristics or did not display differential sensitivity to AD-tau and PSP-tau seeds, whereas the Phospho-Plus substitutions in the R1-R2 and C terminal region showed a clear effect. This could be consistent with the idea that phosphorylation at all sites may not be equivalent in predicting tau aggregation [28]. Specifically, using chemically synthesized K18 fibrils phosphorylated on Ser356, Ser 262 and Ser 258, it was shown that phosphorylation in the microtubule binding domains actually inhibits tau aggregation and PHF assembly [36]. Our data is also consistent with early observations that hyperphosphorylation on several sites, including Ser262, Ser214 and Ser320, prevent PHF assembly [37]. Additionally, phospho-mimicking mutation on Ser305 and Ser 208 also prevented K18-tau induced aggregation in vitro [15, 20]. Interestingly, hibernating mammals undergo extensive phosphorylation on AD-associated epitopes (AT8, AT100, AT180, AT270, PHF-1, and 12E8) without forming tau inclusions, which then gradually reverses when these mammals return to wakefulness [7]. Some reports have shown that phosphorylation of tau on specific epitopes stabilizes it against proteolytic degradation, often a first step towards filament assembly [38, 39]. All of these observations would suggest that phosphorylation of tau may not always lead to misfolding and aggregation, as exemplified by our data that S305E tau is resistant to being templated by AD-tau. A follow-up question stemming from our study and related observations would be if modifying the S305 site in AD patients would halt the progression of self-templating of tau? While this is certainly provocative, caution is warranted as our data shows that phospho-plus substitutions in R1-R2 region of tau leads to decreased microtubule binding. How this would ultimately lead to loss of microtubule plasticity and cellular homeostasis at the cost of reduced tau templating would need to be studied carefully.

PTMs on tau are thought to be related to the creation of tau strains that could determine how tau propagates in the brain [5]. Recent cryo-EM studies have shown that paired helical filaments of tau isolated from patients carry distinctive PTMs that could determine structural specificity during tau filament assembly [40]. Using in silico prediction algorithms, we found that the phospho-site substituted Variant 4 and Variant 5 showed unique fold patterns compared to parent P301L tau (Additional file 1: Fig. S8). Using RoseTTAFold [41], we noticed that both Phospho-Plus Variant 4 and Variant 5 display a more open conformation compared to its parent P301L tau. While Phospho-Null Variant 5 resembled its Phospho-Plus counterpart, Phospho-Null Variant 4 appeared more compact than its Phospho-Plus counterpart (Additional file 1: Fig. S8). Future structure-based studies could address the strain concept of tau as defined by phospho-PTMs using resources generated in this study.

Another possible mechanism could be related to the mutations in the PHF6 region being essential for tau aggregation [42]. The PHF6* (R2: ^275^VQIINK^280^) and PHF6 (R3: ^306^VQIVYK^311^) are two nucleating sequences present within the MTBR. These self-aggregating sequences are within the Variant 4 or just abutting it. Mature tau filaments can be folded in a parallel β-sheet formation through interactions between PHF6–PHF6 and PHF6*–PHF6*, which form extensive homo-steric zipper interface [43]. Recent micro-ED analysis revealed three different interfaces associated with the VQIINK sequence, leading to speculation that these could underlie the molecular basis of tau strains [44]. Insertion of a β-sheet-disrupting proline residue in PHF6 or PHF6* or their deletion suppresses tau aggregation [45], suggesting that the Variant 4 mutations contained within the PHF6* would be similarly able to disrupt the domain-domain interactions, leading to reduced aggregation.

Another key question is whether these phosphorylation sites preceded misfolding or are these added to the tombstone inclusions of tau? A proteomic study by Wesseling and colleagues concluded that there is a sequence of early addition of phospho-PTMs in the PRR domain, followed by acetylation and ubiquitination that progressively leads to tau misfolding and aggregation in AD [14]. Indeed, the selection of phospho-substitution sites in our study was determined from this particular study identifying the early to mid-stage addition of phospho-epitopes on tau. However, it still needs to be determined whether such a gradual ‘processivity’ [14] can be recapitulated in the lifetime of tau molecule. While our study does not address this aspect of tauopathy progression due to its limitation of being in a time-restricted cell culture format, future in vivo studies could address this critical question. Another recent proteomic study identified PTM signatures on soluble tau protein from patients with AD, and related tauopathies, such as CBD, PiD and FTD [13]. Notably, they found consistently increased pS262 (present in Variant 4 in our study) in CBD and FTD patients, while in AD patients, pS262 was always associated with ubiquitinated K267. This could imply that multiple PTMs occurring simultaneously are required for tau to be progressively misfolded in different diseases. Our study did not consider whether specific combinations of phosphorylation epitopes or combinations of different PTMs would yield a disease-specific seeding profile.

In summary, our study is unique as we have considered whether specific phosphorylation patterns on host soluble tau influence its interaction with human brain-derived tau seeds. Our study thus establishes the importance of a phospho-PTM code in tau seeding in two different tauopathies, namely AD and PSP.

## Limitations of our study

There are some limitations in our study, one major point being that this study completely relied on using the FTD-associated P301L mutation as the backbone on which all the phospho-substitutions were created. We tried several iterations of wild type human tau in our model but were unable to reproducibly generate seeded aggregation. Thus, some caution is warranted in extrapolating the implications of our data to AD-typical tau pathology. In addition, while we did not observe any cell death in the phospho-variant seeded cells in the short-term, it is possible that incubation and maturation of seeded tau over longer periods may expose differential cellular vulnerability to individual phospho-specific strains of tau. Such long-term kinetics studies were difficult to execute in our present study due to transience of HEK293T cells and future studies will incorporate primary neuronal cultures to investigate not only cell death but also kinetics of tau aggregation as well as progressive microtubule destabilization in the presence of different phospho-modified tau. Another critical aspect that we did not investigate is whether the seeded aggregates of the phospho-variants are able to induce secondary aggregation. Future experiments will utilize HEK293 cell-generated phospho-mutant aggregates to seed appropriately primed mouse models of tauopathy that could allow us to define the phospho-PTM code in the context of neuroanatomic propagation and strain emergence.

### Supplementary Information

**Additional file 1.** Supplementary Figures and Figure Legends.

**Additional file 2.** Supplementary Tables and descriptions.

## Data Availability

All data generated or analysed during this study are included in this published article [and its supplementary information files].

## Abbreviations

AD: Alzheimer’s disease
AD-tau: Sarkosyl (detergent) insoluble tau from AD patient brain
CBD: Cortico-basal degeneration
FRET: Fluorescence resonance energy transfer
FTD: Fronto-temporal dementia
NFT: Neurofibrillary tangle
Phospho-Null: Phosphorylation ablating mutations (Ser/Thr → Ala)
Phospho-Plus: Phosphorylation mimicking mutations (Ser/Thr → Glu)
Phospho-tau: Phosphorylated tau
PiD: Pick’s disease
p-tau: AT8 epitope phosphorylated tau
PRR: Proline-rich region
PSP: Progressive supranuclear palsy
PSP-tau: Sarkosyl (detergent) insoluble tau from PSP patient brain
Phospho-PTM: Phosphorylation as a post translational modification
PTM: Post translational modification
t-tau: Total tau (non-phosphorylated)
