# Supplementary material for: Human tauopathy strains defined by phosphorylation in R1-R2 repeat domains of tau
Source: Acta Neuropathol Commun. 2023 Oct 27;11:172. doi: 10.1186/s40478-023-01664-0 (PMC10612232; doi:10.1186/s40478-023-01664-0)
Supplement: Supplementary file 1 — Additional file 1. Supplementary Figures and Figure Legends. [file 40478_2023_1664_MOESM1_ESM.docx]

**Report: Human tauopathy strains defined by phosphorylation in R1-R2 repeat domains of tau**

Ethan D Smith, Quan Vo, Benoit I Giasson, David R Borchelt, Stefan Prokop, Paramita Chakrabarty.

**Supplemental Information - Supplemental Figures with Title and Legends; Supplemental Table Title and Legends.**

**
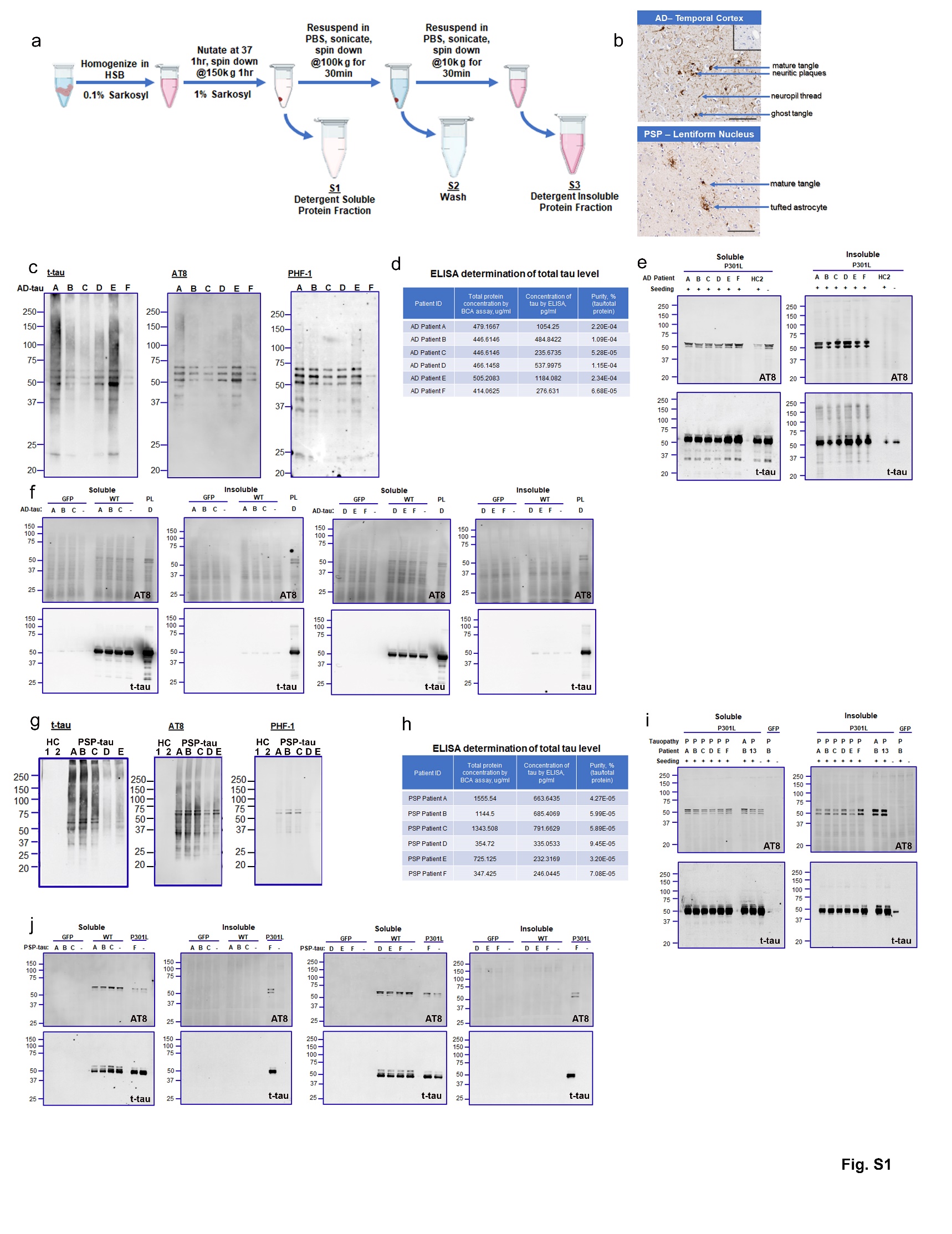
**

**Figure S1: Validation of tau in sarkosyl-extracted human brains.**

a, Schematic of the isolation protocol of sarkosyl-insoluble tau seeds used for this study. b, Representative image of temporal cortex from a typical AD and Lentiform Nucleus of a PSP patient in the study cohort stained with AT8 antibody. c-f, Representative western blots of sarkosyl-insoluble AD-tau isolated from different AD patients stained for total tau (t-tau), AT8 and PHF1 (c). ELISA of sarkosyl-insoluble AD-tau isolated from different AD patients detecting total tau (d). AD-tau (+) isolated from Patients A through F or from age-matched cognitively normal individual (HC2) were used to seed HEK293T cells transfected with P301L tau (e). Cells were lysed and fractionated into detergent-soluble and detergent-insoluble lysates and probed for AT8 or total tau (e). As controls, AD-tau from Patients A through F were used to seed HEK cells expressing GFP or 0N/4R wild type tau (f). g-j. Representative western blots of sarkosyl-insoluble PSP-tau isolated from different PSP patients stained for total tau (t-tau), AT8 and PHF1 (g). ELISA of sarkosyl-insoluble AD-tau isolated from different PSP patients detecting total tau (h). Establishing seeding ability of sarkosyl-insoluble PSP-tau on P301L tau (i). PSP-tau (+) isolated from Patients A through F and #13 were used to seed HEK293T cells transfected with P301L tau. Cells were lysed and fractionated into detergent-soluble and detergent-insoluble lysates and probed for AT8 or total tau (i). As controls, PSP-tau from Patients A through F were used to seed HEK cells expressing GFP or 0N/4R wild type tau (j). Relative molecular masses are indicated on the left of each blot.

**
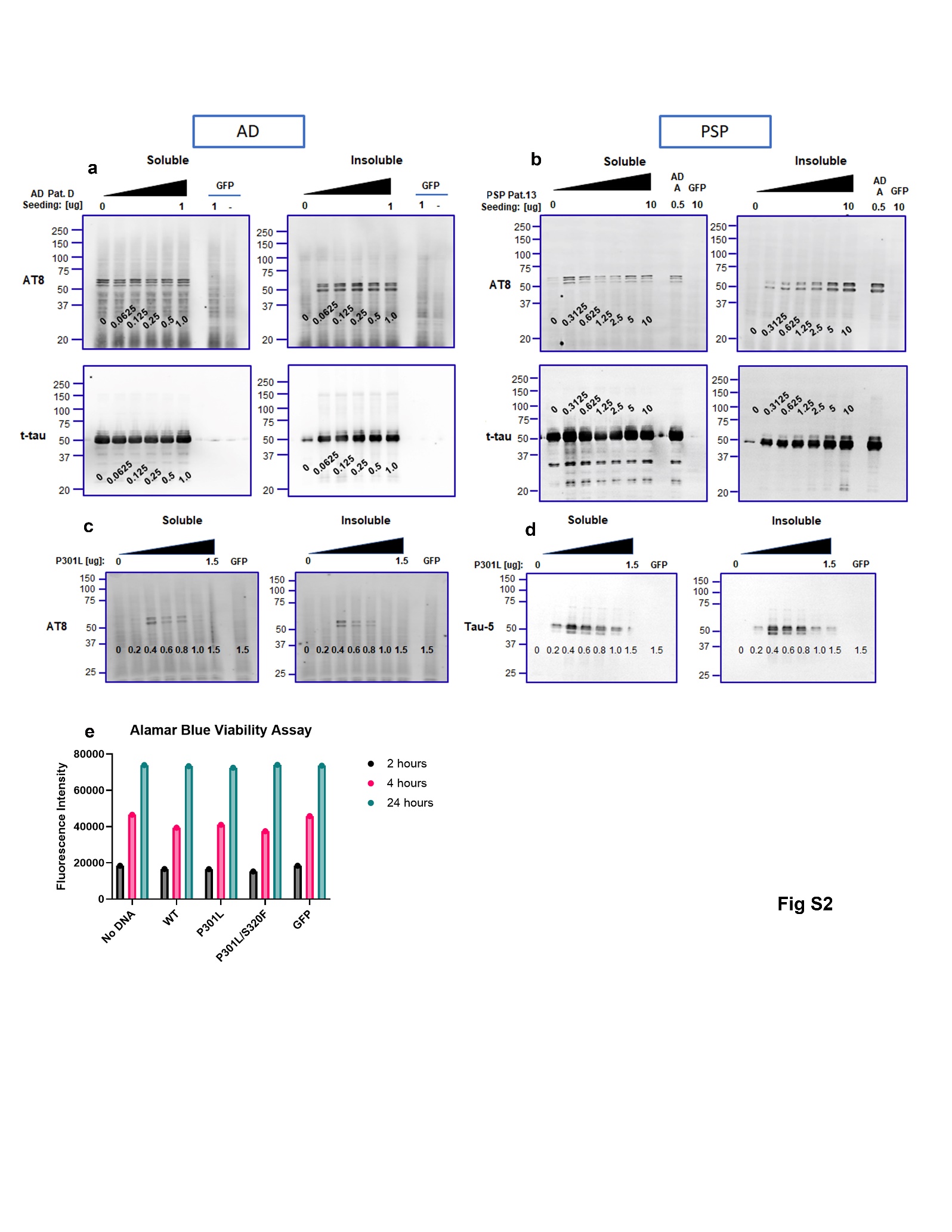
**

**Figure S2: Optimization of seeding threshold by varying amount of brain-derived seeds and acceptor tau DNA in HEK293 seeding assay.**

a-b. Representative western blot of HEK293T cells transfected with either P301L or GFP and seeded using AD-tau derived from Patient D (a) or PSP-tau derived from Patient 13 (b). Cells were lysed and extracted in detergent, yielding detergent-soluble and detergent-insoluble tau. AD-tau seeds were serially diluted from 1µg to 0.0625µg while PSP-tau seeds 10µg to 0.3125µg and used for seeding assay. Negative GFP control was seeded with 1µg of patient D AD-tau or 10µg of patient 13 PSP-tau. In PSP-tau seeding blots, an additional control of AD patient A (0.5µg AD-tau) was used. n=2 experimental replicates. c-d. To optimize 0N4R P301L DNA amount to be used for transfection, P301L DNA was diluted in a step-wise fashion and seeded with K18 tau fibrils (1µM). Cells were lysed and extracted in detergent, yielding detergent-soluble and detergent-insoluble tau detected using AT8 and total tau antibody (t-tau, Abcam Cat# ab254256 or Tau5). n=2 experimental replicates. e. AlamarBlue cell viability was conducted on HEK293 cells transfected with different plasmids: WT 0N4R tau, P301L 0N4R tau, P301L/S320F 0N4R tau, and GFP. Measurements were taken at 3 different time points following seeding with AD-tau.

**
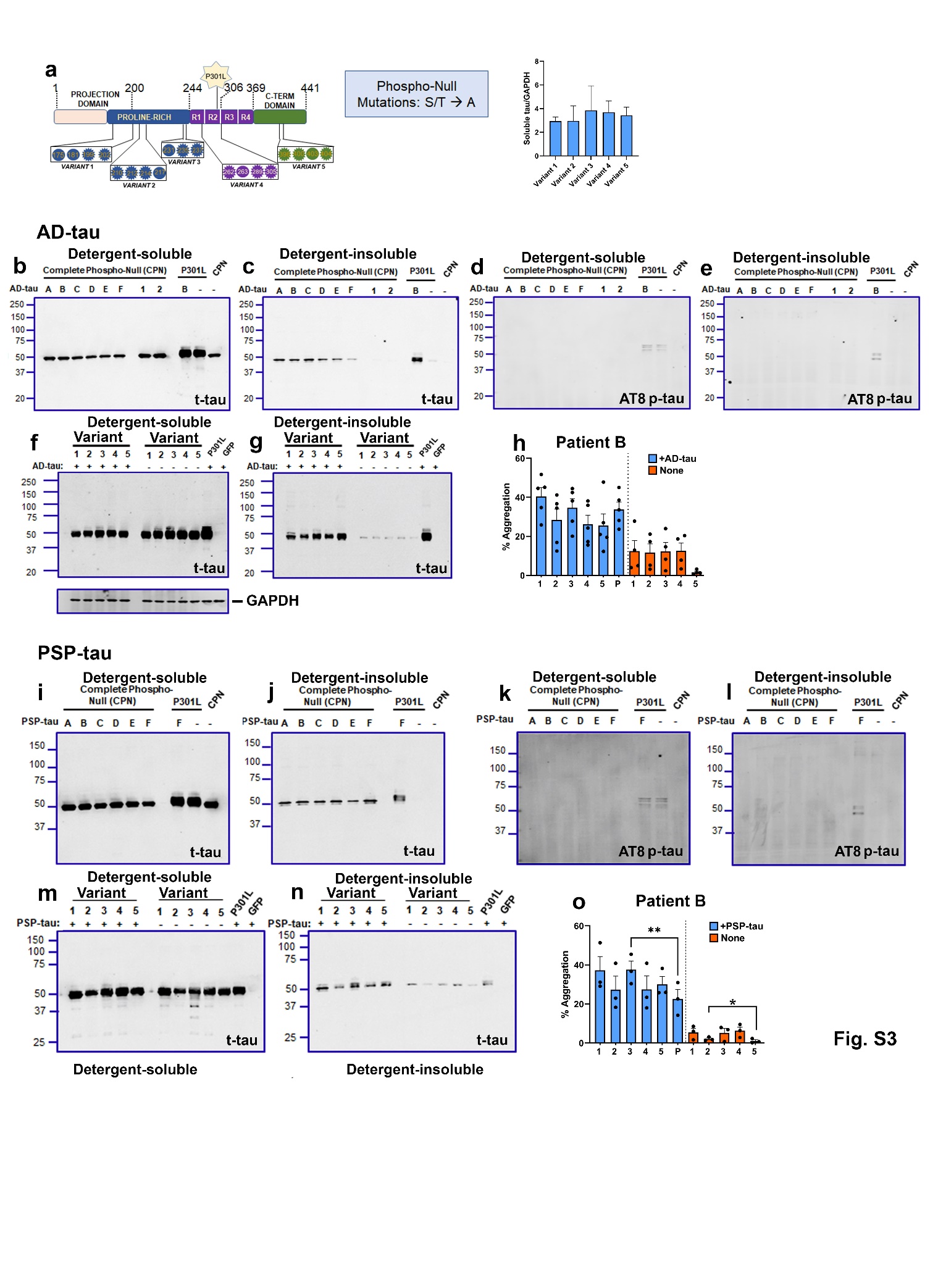
**

**Figure S3: Ablating phosphorylation on select sites does not significantly affect tau aggregation in AD-tau seeded or PSP-tau seeded cells.**

a, Schematic depiction of phospho-deficient Ser/Thr → Ala tau variants generated on human 0N/4R P301L mutant tau. All numbers correspond to 2N/4R tau. The graph shows relative expression levels of different Phospho-Null variants calculated from unseeded variant protein level normalized to GAPDH. N=2/variant. b-e, Representative immunoblot of the Complete Phospho-Null (CPN) construct with all 19 sites mutated to Ala and seeded with AD Patients A-F or two healthy controls (1, 2) shown. P301L tau seeded with Patient B or left unseeded are also shown. Total tau (t-tau) and AT8-tau (p-tau) shown from detergent-soluble (b, d) or insoluble (c, e) cell lysates. f-k, Patient B AD-tau (+) or unseeded (-) HEK293T cells transfected with different tau variants were fractionated into detergent-soluble and detergent-insoluble lysates and probed for total tau (f-h). Quantitation of % aggregation (insoluble/[soluble+insoluble]*100) for each variant are shown (h). i-o, Representative immunoblot of the Complete Phospho-Null (CPN) construct with all 19 sites mutated to Ala and seeded with PSP Patients A-F shown. P301L tau seeded with Patient F or left unseeded are also shown. Total tau (t-tau) and AT8-tau (p-tau) shown from detergent-soluble (I, k) or insoluble (j, l) cell lysates. m-o, Patient B PSP-tau (+) or unseeded (-) HEK293T cells transfected with different tau variants were fractionated into detergent-soluble and detergent-insoluble lysates and probed for total tau (m-n). Quantitation of % aggregation (insoluble/[soluble+insoluble]*100) of all variants seeded by PSP Patient B are shown (o). GAPDH is the loading control for detergent-soluble fraction. Broken lines (h, o) depict statistical tests done separately within groups of seeded or non-seeded tau variants. Numbers 1-5 denote the tau Variants (f-k; p-u); ‘P’, P301L tau; ‘G’, GFP; ‘ND’, not detected. Relative molecular masses (kDa) are indicated on the left of each blot. N=3 experimental replicates. 1-way ANOVA with Dunnett’s test; **p<0.01; *p<0.05.

**
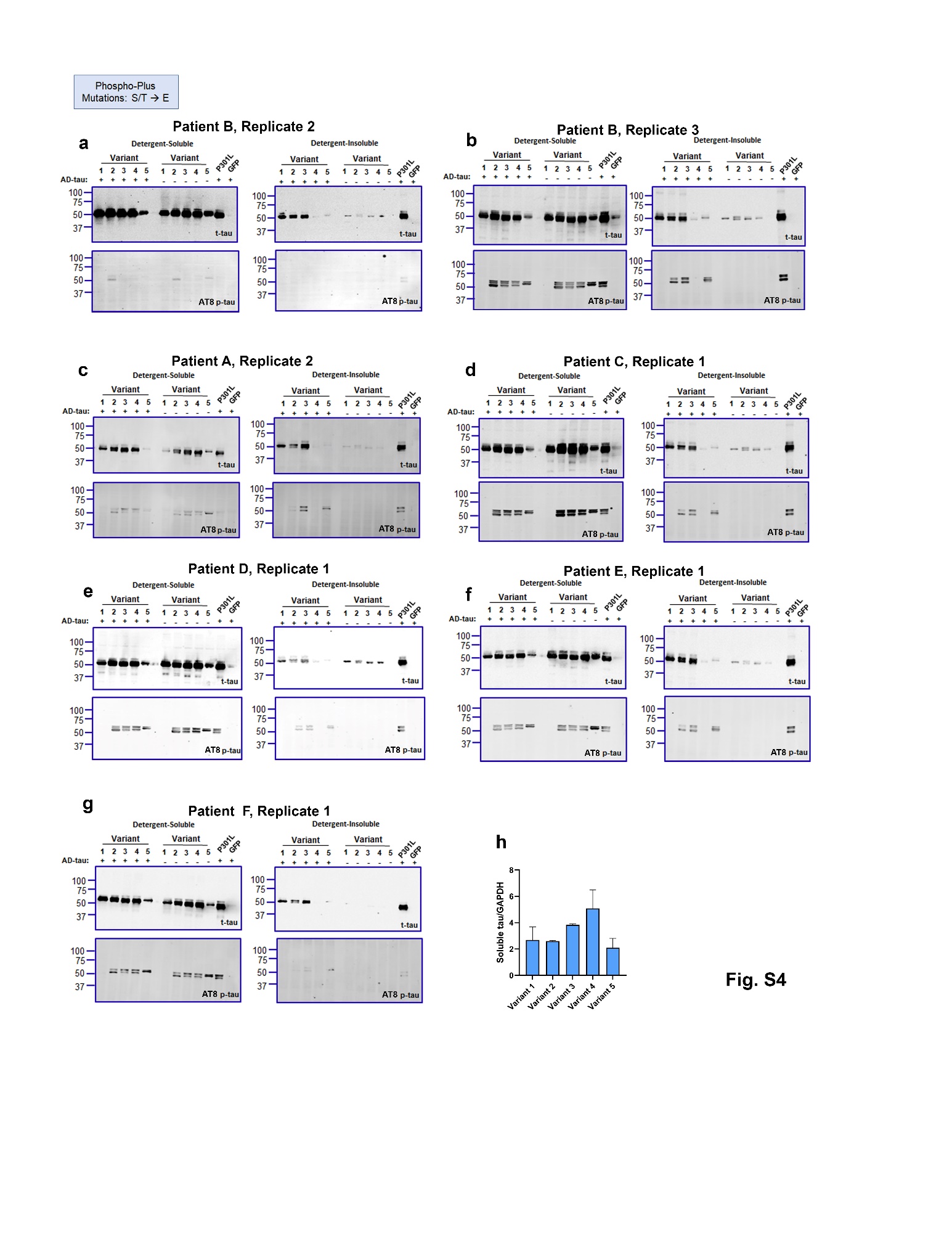
**

**Figure S4.** **Additional western blots in support of Fig. 1.**

Additional replicates of AD Patient B (depicted in Fig. 1) and representative western blots for AD Patients A, C, D, E and F are shown following AD-tau seeding of phospho-plus Ser/Thr → Glu tau variants generated on human 0N/4R P301L mutant tau backbone. h. Relative expression levels of different Phospho-Plus variants was calculated from unseeded variant protein level normalized to GAPDH. N=2/variant.


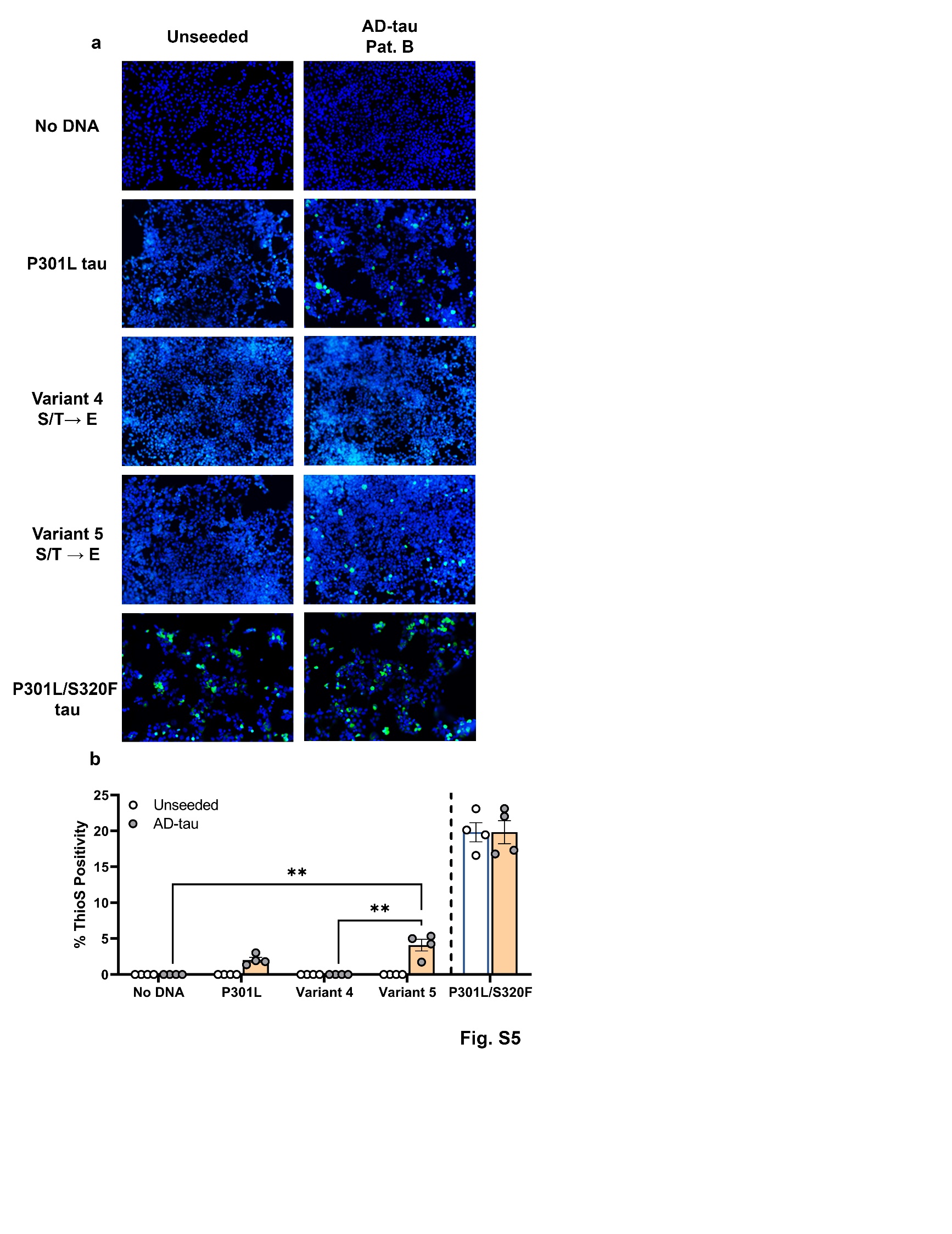


**Figure S5: Confirmation of inclusion formation in AD-tau seeded HEK293T cells.**

a, Representative 20x immunofluorescence images of HEK293T cells transfected with plasmids expressing no tau DNA, P301L tau, phospho-plus variant 4, phospho-plus variant 5, or P301L/S320F 0N4R tau and seeded with 0.5µg of AD-tau from AD Patient B. P301L/S320F tau is a self-aggregating tau construct and is the positive control for inclusions. The cells were stained with Thioflavin S (green) and DAPI (nucleus, blue). b, Quantification of %ThioS positivity for all conditions as a ratio of number of ThioS positive cells to DAPI positive cells. n=4 images (fov) per condition; 2 wells per condition. 2-way Anova, **p<0.01. Broken line depicts that data from P301L/S320F tau were excluded from statistical analysis.

**
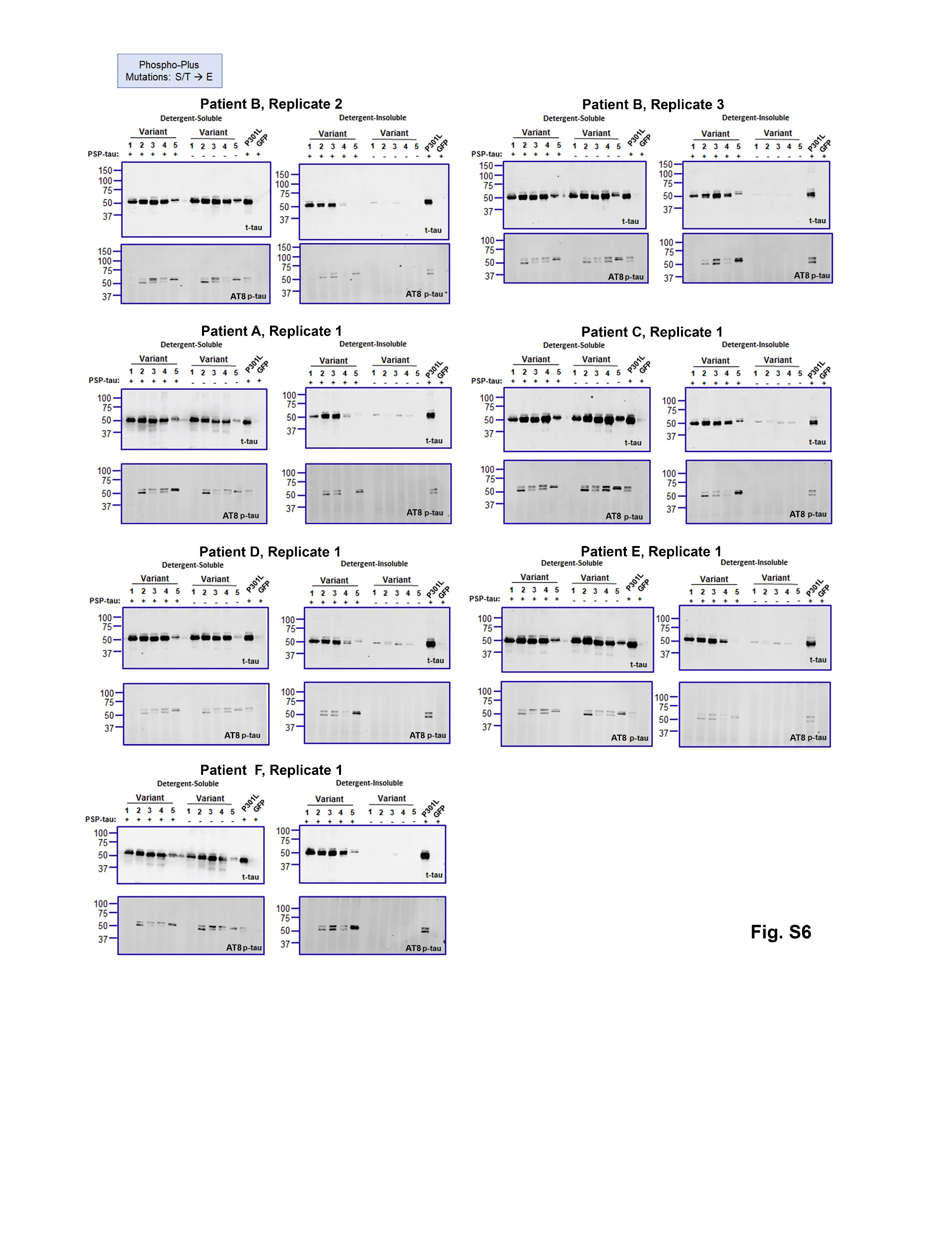
**

**Fig. S6: Additional western blots in support of Fig. 2.**

Additional replicates of PSP Patient B (depicted in Fig. 2) and representative western blots for PSP Patients A, C, D, E and F are shown following PSP-tau seeding of phospho-mimicking Ser/Thr → Glu tau variants generated on human 0N/4R P301L mutant tau backbone.

**
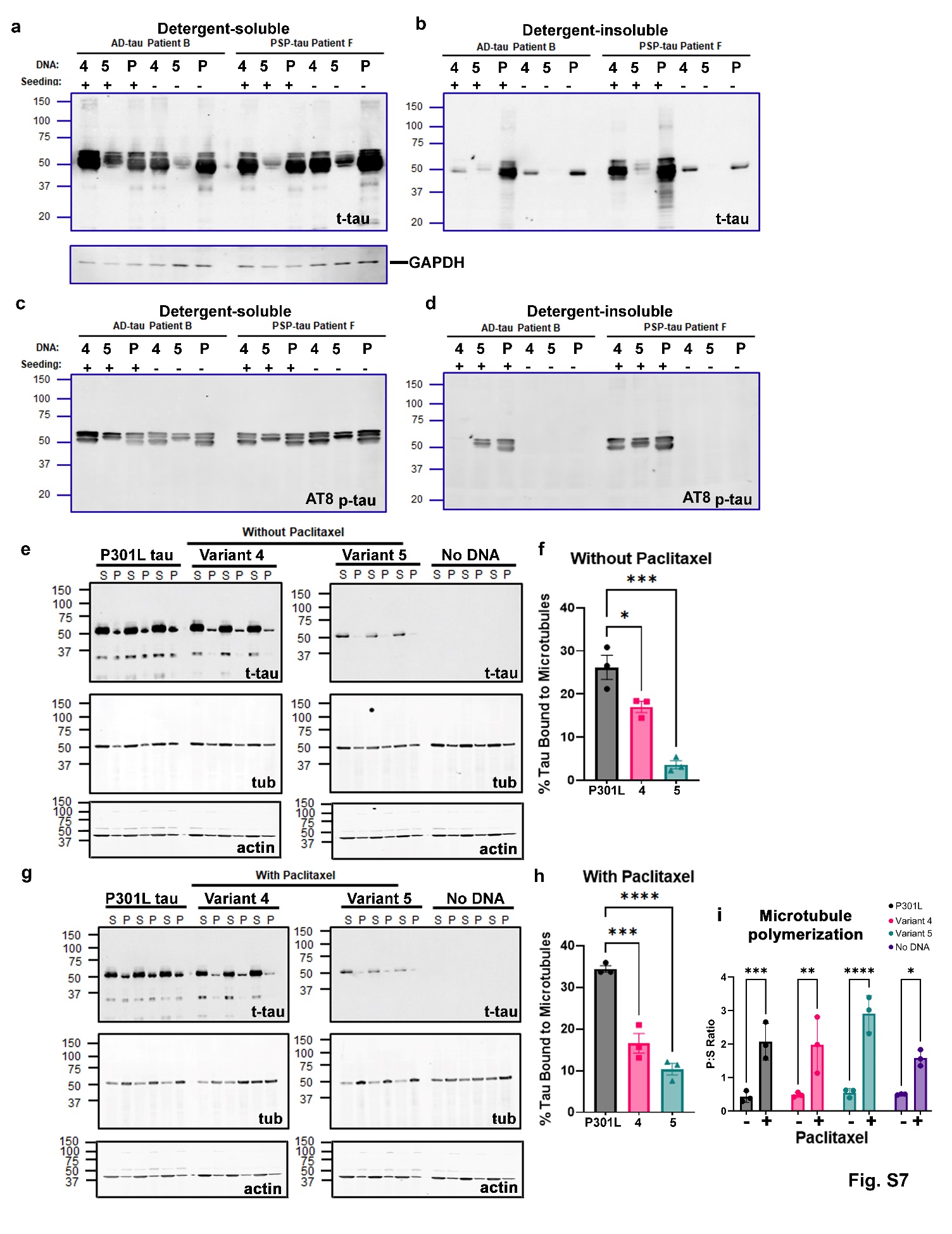
**

**Fig. S7. Phospho-plus Variant 4 and Variant 5 shows reduced microtubule binding.**

**a-d.** HEK293T cells were transfected with phospho-plus variant 4, phospho-plus variant 5, or P301L tau and seeded with 0.5µg of AD-tau or PSP-tau as indicated. Cells were fractionated into detergent-soluble and insoluble fractions and tested for presence of total tau and AT8 p-tau. GAPDH is the loading control. 4, Variant 4; 5, Variant 5; P, P301L tau. **e-h.** Cell-based microtubule binding assay performed with HEK293T cells transfected with P301L tau, and phospho-plus variants 4 or 5, as well as a “No DNA” negative control that was treated with (g, h) or without (e, f) paclitaxel. Antibody specific for β-tubulin was used to detect polymerized microtubule. i. Extent of microtubule polymerization (P:S ratio calculated from panels e and g) depicted for different tau variant expressing conditions in the presence or absence of paclitaxel. S= supernatants; P= pellet fractions; tub= β tubulin. The relative molecular masses of protein markers are indicated on the left. n=3 replicates; 1-way ANOVA with Dunnett’s test; ****p<0.0001; ***p<0.001; *p<.05.

**
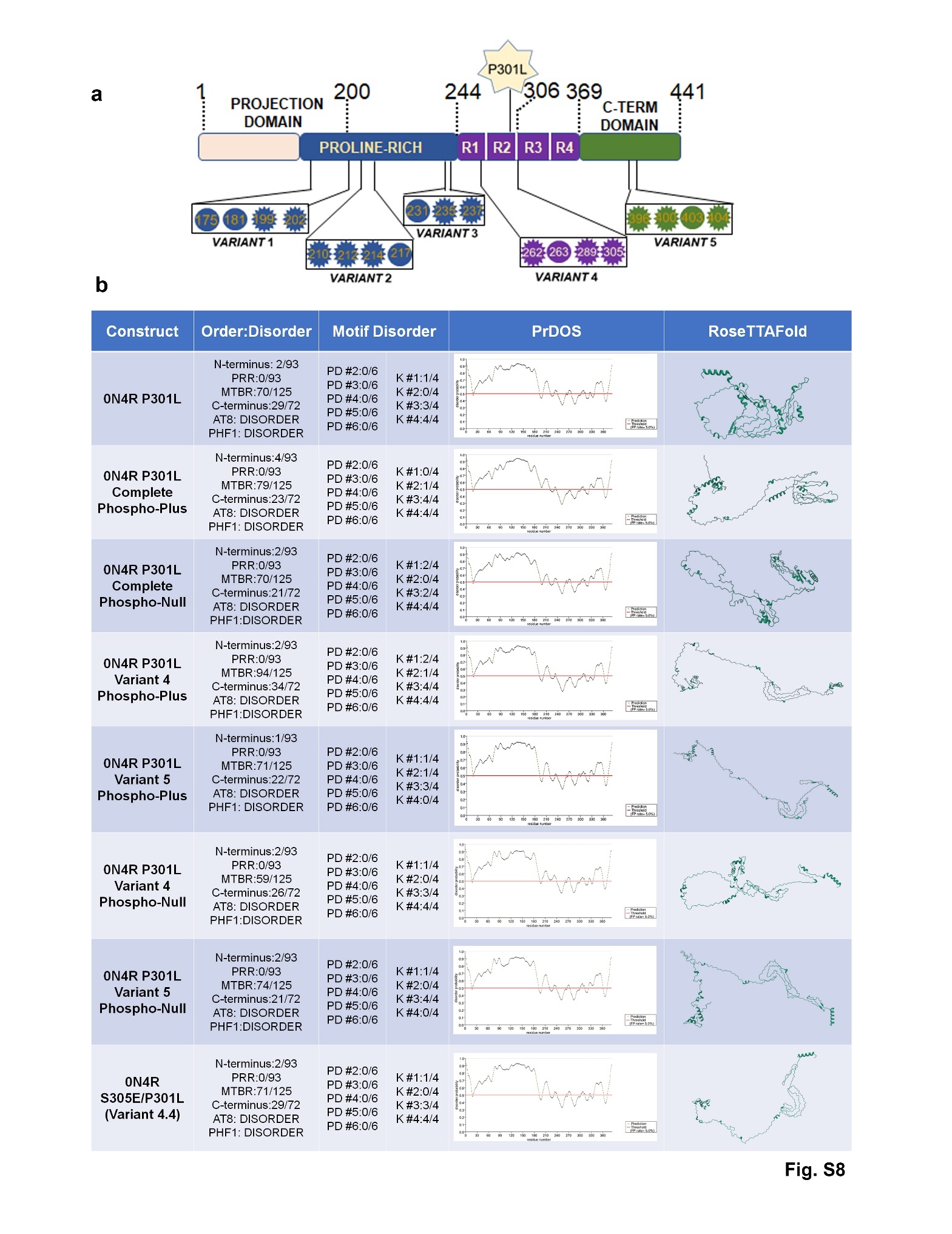
**

**Figure S8. In silico structural prediction of tau variants used in the study.**

a. Schematic depiction of phospho-substituted tau variants generated on human 0N/4R P301L mutant tau. All numbers correspond to 2N/4R tau. R1-R4 depict microtubule-binding repeat regions. Rounded circles are Ser residues and serrated circles are Thr residues. b. In silico predictions were conducted using metaPrDOS which combines software integrating predictions of regional disorder from amino acid sequence. The disorder plot shows regions of order (below the red line) and regions of disorder (above the red line). RoseTTAFold is a software which predicts protein conformation based on both chemical and physical relationships. Modeling of Phospho-Plus Variant 4 and Phospho-Plus Variant 5 representing MTBR and C-terminal tau mutants display a more open conformation compared to parent P301L tau, suggesting these mutations might play a role in conformational changes in tau.

**Supplementary Table Legends.**

**Supplementary Table S1. Demographics and diagnoses of the patient cohorts used in this study.** Case IDs correspond to individually characterized brains in the University of Florida Neuromedicine Human Brain and Tissue Bank. Experimental Group denotes the designation for this study. NP Dx1, primary diagnosis based on neuropathology; NP Dx2 and NP Dx3, secondary diagnoses based on neuropathology; Thal phase, burden of immunostained amyloid deposits in cortical and subcortical area; Braak stage, CERAD score, neuritic plaque frequency; AD, Alzheimer's disease; ARTAG, Aging-related tau astrogliopathy; CAA, cerebral amyloid angiopathy; HC, healthy control with no dementia; LATE, limbic-predominant age-related TDP-43 encephalopathy.

**Supplementary Table S2. Antibodies used in this study.**
