# Supplementary material for: Human tauopathy strains defined by phosphorylation in R1-R2 repeat domains of tau
Source: Acta Neuropathol Commun. 2023 Oct 27;11:172. doi: 10.1186/s40478-023-01664-0 (PMC10612232; doi:10.1186/s40478-023-01664-0)
Supplement: Supplementary file 2 — Additional file 2. Supplementary Tables and descriptions. [file 40478_2023_1664_MOESM2_ESM.pdf]

Table S1. Demographics and diagnoses of the patient cohorts used in this study.Individually characterized brains in the University of Florida Neuromedicine Human Brain and Tissue Bank were used in the study. Experimental Group denotes the designation for this study. NP Dx1, primary diagnosis based on neuropathology; NP Dx2 and NP Dx3, secondary diagnoses based on neuropathology; Thal phase, burden of immunostained amyloid deposits in cortical and subcortical area; Braak stage, CERAD score, neuritic plaque frequency; AD, Alzheimer's disease; ARTAG, Aging-related tau astrogliopathy; CAA, cerebral amyloid angiopathy; CVD, cardiovascular disease; HC, healthy control with no dementia; LATE, limbic-predominant age-related TDP-43 encephalopathy; PSP, Progressive supranuclear Palsy. N/A: Not applicable; ND, not done.

Experimental Group	Post-mortem Diagnosis	NP Dx1	NP Dx2	NP Dx3	Thal stage	Braak stage	CERAD score	APOE genotype	Sex	Age	PMI
AD-A	AD	AD high	CAA widespread, mild	N/A	4	V	frequent	3/4	m	77	5
AD-B	AD	AD high	CAA widespread, moderate	N/A	5	VI	frequent	3/4	m	63	2
AD-C	AD	AD high	CAA widespread, mild to moderate	LATE stage 2	4	VI	frequent	3/4	f	82	7
AD-D	AD	AD high	CAA (moderate to severe)	Acute microinfarcts	4	VI	frequent	3/4	m	73	6
AD-E	AD	AD high	CAA widespread, moderate	N/A	5	VI	frequent	3/4	m	59	7
AD-F	AD	AD high	CAA widespread, moderate	N/A	5	VI	frequent	3/3	m	83	7
LN-13	PSP	PSP	AD low	N/A	3	I	none	3/3	f	77	10
LN-A	PSP	PSP	AD low	CAA focal, mild to moderate	3	I	none	ND	m	70	19.5
LN-B	PSP	PSP	AD intermediate	CAA widespread, severe	5	III	moderate	4/4	m	72	27
LN-C	PSP	PSP	LATE Stage2	AD low	1	II	none	3/3	m	77	12
LN-D	PSP	PSP	AD low	ARTAG	3	I	none	3/3	m	78	3
LN-E	PSP	PSP	LATE Stage 1	N/A	0	0	none	3/3	f	70	18
LN-F	PSP	PSP	AD low	CAA focal, mild	1	II	sparse	3/3	m	76	6
HC-1	Control	N/A	N/A	N/A	0	0	none	3/4	f	55	12
HC-2	Low AD	AD low	CVD	N/A	1	II	none	ND	m	88	4

Table S2. Antibodies used in this study

Antibody name	Specificity	Host Species	Dilutions	Source
Recombinant anti-tau	Mouse and human tau	Rabbit monoclonal	1:10000 (WB)	Abcam Cat# ab254256, RRID:AB_2894402
Tau-5	Mouse, human, rat, bovine, and sheep tau	Mouse monoclonal	1:1000 (WB)	Innovative Research Cat# AHB0042, RRID:AB_1502093
DAKO	Human tau	Rabbit polyclonal	1:2500 (IHC)	Agilent Cat# A0024, RRID:AB_10013724
AT8	pSer202/pT205	Mouse monoclonal	1:1000 (WB)	Thermo Fisher Scientific Cat#MN1020, RRID:AB_223647
PHF1	pSer396/pSer404	Mouse monoclonal	1:1000 (WB)	Gift from Dr. Peter Davies (Greenberg et al., 1992; RRID:AB_2313687)
AT270	pThr181	Mouse monoclonal	1:1000 (WB)	Thermo Fisher Scientific Cat#MN1050, RRID:AB_223651
AT100	pThr212/pSer214	Mouse monoclonal	1:1000 (WB)	Thermo Fisher Scientific Cat# MN1060, RRID:AB_223652
AT180	pThr231	Mouse monoclonal	1:1000 (WB)	Thermo Fisher Scientific Cat# MN1040, RRID:AB_223649
pSer262	pSer262	Rabbit polyclonal	1:1000 (WB)	Thermo Fisher Scientific Cat# 44-750G, RRID:AB_2533743
pSer422	pSer422	Rabbit polyclonal	1:1000 (WB)	Thermo Fisher Scientific Cat# 44-764G, RRID:AB_2533748
Tau C3	Tau cleaved at Asp421 and Asp422	Mouse monoclonal	1:1000 (WB)	Thermo Fisher Scientific Cat# AHB0061, RRID: AB_2536237
GAPDH	GAPDH	Rabbit monoclonal	1:1000 (WB)	Abcam Cat# ab181602, RRID:AB_2630358
TUB 2.1	β-tubulin	Mouse monoclonal	1:1000 (WB)	Sigma-Aldrich Cat# T4026, RRID:AB_477577
actin	β-actin	Mouse monoclonal	1:1000 (WB)	Sigma-Aldrich Cat# A5441, RRID:AB_476744
